# Broadband light trapping strategies for quantum-dot photovoltaic cells (>10%) and their issues with the measurement of photovoltaic characteristics

**DOI:** 10.1038/s41598-017-17550-4

**Published:** 2017-12-12

**Authors:** Changsoon Cho, Jung Hoon Song, Changjo Kim, Sohee Jeong, Jung-Yong Lee

**Affiliations:** 1 Graduate School of Energy, Environment, Water, and Sustainability (EEWS), Korea Advanced Institute of Science and Technology (KAIST), Daejeon, 34141 Republic of Korea; 2 Korea Institute of Machinery & Materials (KIMM), Daejeon, 34103 Republic of Korea

## Abstract

Bandgap tunability and broadband absorption make quantum-dot (QD) photovoltaic cells (PVs) a promising candidate for future solar energy conversion systems. Approaches to improving the electrical properties of the active layer increase efficiency in part. The present study focuses on optical room for enhancement in QD PVs over wide spectrum in the near-infrared (NIR) region. We find that ray-optical light trapping schemes rather than the nanophotonics approach may be the best solution for enhancing broadband QD PVs by suppressing the escape probability of internal photons without spectral dependency. Based on the theoretical study of diverse schemes for various bandgaps, we apply a V-groove structure and a V-groove textured compound parabolic trapper (VCPT) to PbS-based QD PVs along with the measurement issues for PVs with a light scattering layer. The efficiency of the best device is improved from 10.3% to 11.0% (certified to 10.8%) by a V-groove structure despite the possibility of underestimation caused by light scattering in small-area devices (aperture area: 0.0625 cm^2^). By minimizing such underestimation, even greater enhancements of 13.6% and 15.6% in short circuit current are demonstrated for finger-type devices (0.167 cm^2^ without aperture) and large-area devices (2.10 cm^2^ with an aperture of 0.350 cm^2^), respectively, using VCPT.

## Introduction

Low-cost thin-film photovoltaic cells (PVs) have recently shown a dramatic increase in power conversion efficiency (PCE). Securing light absorption has been a significant issue for thin-film PVs with a nanometer-scale optical path length limited by their poor charge carrier mobility. Along with the development of novel materials, recent cutting-edge organic and perovskite PVs show a high device absorption of >80% and >90%, respectively, for photons within their bandgaps (1.5–2 eV)^1–5^. For those PVs, nanophotonical approaches adopting metal nanoparticles^6–12^ or nanopatterning the interfaces^4,5,11–19^ have been shown to induce surface plasmon resonance (SPR) and effectively suppress optical loss at specific less-absorptive wavelength regions such as band-edge^4,13,14^.

In addition to high absorption, broadening the absorption spectrum is another issue for thin-film PVs to achieve ultrahigh efficiency, whereas IR photons below 1.5 eV account for ~40% of total solar energy^20^. Accordingly, quantum-dot (QD) PVs are attracting great attention owing to their bandgap tunability and multiple exciton generation (MEG) effect, possibly breaking the Shockley–Queisser limit^21,22^ of PCEs^23–26^. Rapid advancement of QD PVs in recent years has yielded high PCEs comparable to those of organic PVs (OPVs) (>10%)^27–32^; PCEs of crystalline silicon (c-Si) PVs (>20%) and the Shockley–Queisser limit (>30%) are the next milestones. QDs used in QD PVs have relatively low bandgaps below 1.5 eV and thus a broad absorption spectrum around the near-IR (NIR) region. However, whereas c-Si PVs typically have a short-circuit current density (*J*_sc_) greater than 40 mA/cm^2^ by absorbing light below 1100 nm^33,34^, *J*_sc_ of QD PVs with a similar spectrum range is typically near or below 30 mA/cm^2^ ^27–30^ owing to the low extinction coefficient for NIR, causing broadband optical loss. The optical properties of low-bandgap QD PVs with a broadband loss have been rarely discussed; a customized design rule is needed for light trapping schemes that outperform previous nanophotonical approaches^8,35–37^ that target specific wavelengths.

Ray-optical approaches can resolve these optical issues, which have been ignored in thin-film PVs for a while. Previous studies for ray-optical light trapping can be classified into external attachment of textured surfaces^12,38–46^ or concentrator arrays^4,12,47–50^ and nonplanar configurations such as double parabolic trapper (DPT)^49^ or folded configurations^51–54^. Although these schemes have been less spotlighted in thin-film PVs with high bandgaps, they could provide broadband optical gains in QD PVs with low bandgaps if applied appropriately.

**Figure 1:**
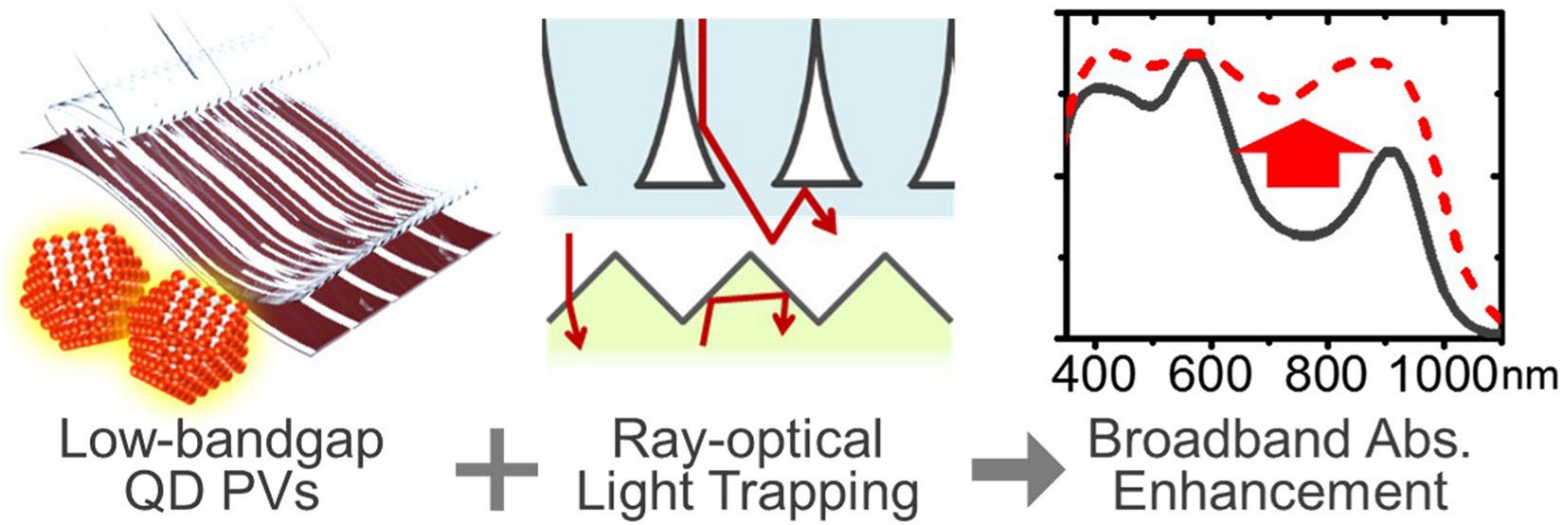
Illustration of ray-optical light trapping for enhancing QD PV absorption.

The present study aims at maximizing QD PV efficiencies by adopting ray-optical strategies as depicted in Fig. 1. Light trapping for QD PVs are investigated with both theoretical studies and experimental demonstration.

## Results and Discussion

**Figure 2:**
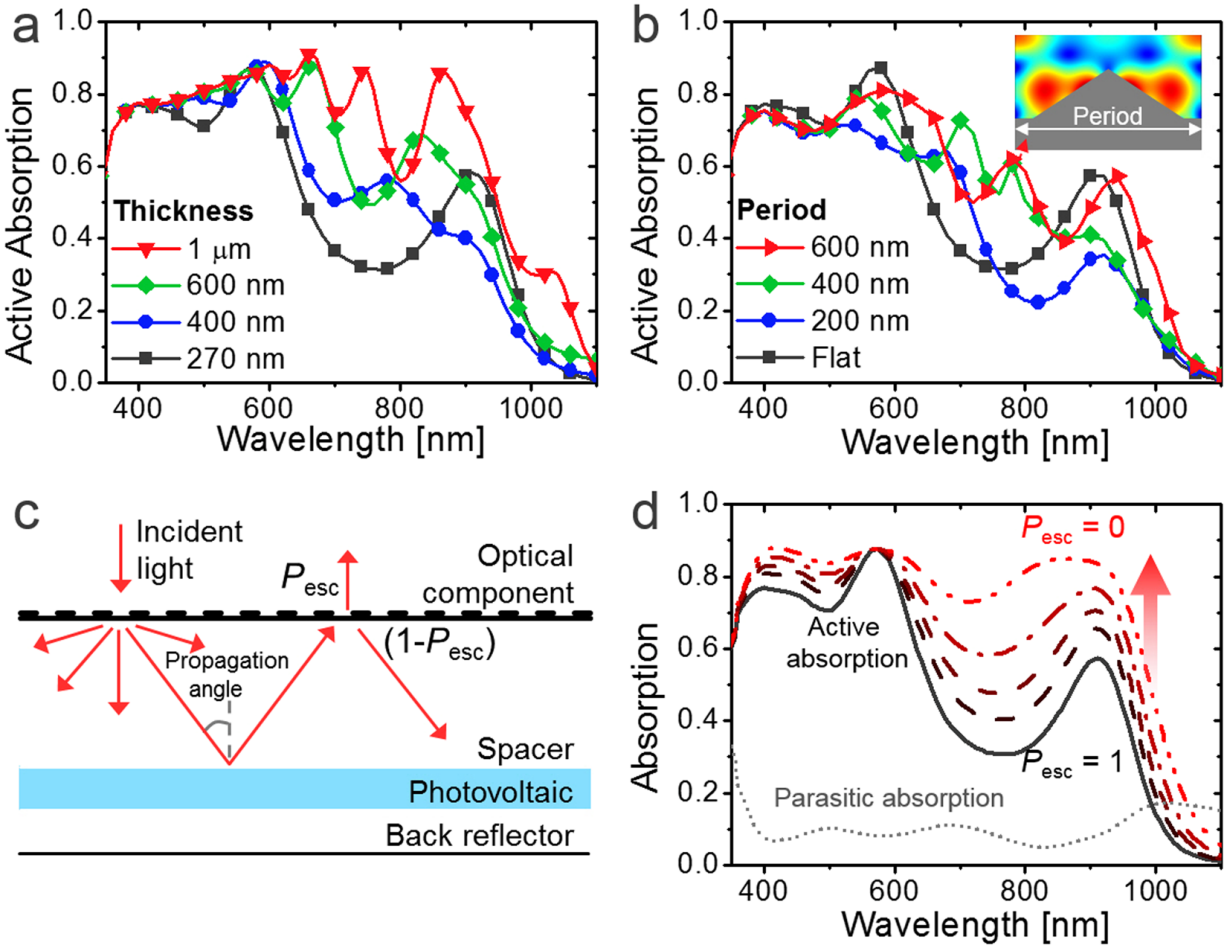
(**a**) Active absorption spectra of QD PV with various active layer thicknesses. (**b**) Active absorption spectra of nanopatterned QD PV with various periods depicted in the inset. (**c**) Schematic expression of a PV system composed of an optical component, a spacer, PVs, and a back reflector. (**d**) Active and parasitic absorption spectra of QD PV according to *P*_esc_ at a normal propagation angle.

In Fig. 2a, a black line represents the PbS QD layer absorption, calculated with a transfer-matrix formalism (TMF)^55,56^, in the PV structure of indium tin oxide (ITO, 75 nm)/ZnO (50 nm)/PbS QD (270 nm)/Au. The bandgap (i.e. the tail of the absorption band) of PbS QD was calculated to be 1.27 eV, where its first excitonic peak was observed near ~850 nm in solution and ~900 nm in a solid state (Figures S1 and S8). Increasing the PbS layer thickness is an obvious solution to reduce optical loss in the active spectrum. As the thickness increases to 400 nm, 600 nm, and 1 μm, the overall photon absorption for the AM 1.5 G spectrum (i.e. ∫ [active absorption × *S*(AM 1.5 G)] d*λ*) is shown to increase by 9.0%, 24%, and 42%, respectively, as exhibited in Fig. 2a. It should be noted that the absorption with increased thickness might be even reduced at some region as the internal electromagnetic field distribution is changed^13,55,56^. However, whereas the recent cutting-edge QD PVs tend to be focused in improving *J*_sc_ with thicker active layers, reduction in the electrical properties cannot be fully avoided for thick layers owing to the limited carrier mobility^28,29,57–59^. Adoption of proper light trapping schemes can be an alternative solution to relieve such a tradeoff and increase light absorption without deteriorating electrical properties.

Figure 2b shows the QD layer absorption of the device (PbS QD bandgap: 1.27 eV, thickness: 270 nm), calculated via the finite element method (FEM)^4,13,14,60^, with and without periodic triangular metal nanogratings with a height of 150 nm and various periods as shown in the inset. Periodic boundary condition was assumed and the TE-polarized and TM-polarized results were averaged. The absorption is shown to increase near the resonant wavelengths of SPR and the conditions can be simply changed by varying the period. However, there is no structure that is beneficial for all the wavelengths since single SPR is not sufficient to cover the broadband absorption spectrum and even reduces absorption outside the resonant wavelengths. For this reason, we focus on investigating ray-optical approaches with a broadband light trapping effect rather than nanophotonics for QD PVs.

We generalize typical ray-optical schemes by dividing them into (i) optical components on the top surface; (ii) an optical spacer for ray propagation, usually a glass substrate; (iii) photovoltaic multilayers to absorb photons; and (iv) a back reflector, usually a metal electrode, as shown in Fig. 2c. Contrary to bulk-type PVs, which have a thick active layer as a spacer, the optical spacer and the photovoltaic layers are separated in thin-film PVs, which is why the oblique propagation of light does not necessarily increase the absorption^49^. Whereas the escape probability (*P*_esc_)^4,13,45^ of the internal photons after the first bounce on the photovoltaic layer is almost one for planar systems, it can be suppressed by proper optical components directing them toward the photovoltaic layers again, as expressed in Fig. 2c. The expected number of multiple bounces becomes *N*_bounce_ = 1/*P*_esc_^4,13,45^.

Figure 2d shows the active absorption spectra of QD PVs for various *P*_esc_ values with an assumed fixed propagation angle of zero. The active absorption increases as *P*_esc_ decreases and photons bounce more on the active layers (refer to SI for the calculation method). The maximum active absorption with perfect light trapping (*P*_esc_ = 0) is limited by the ratio of active absorption to the amount of parasitic absorption of nonactive layers such as the metal electrode. If the optical components are made of microstructures, *P*_esc_ does not depend on the wavelength, and a light trapping effect can be uniformly achieved for a broadband spectrum.

**Figure 3:**
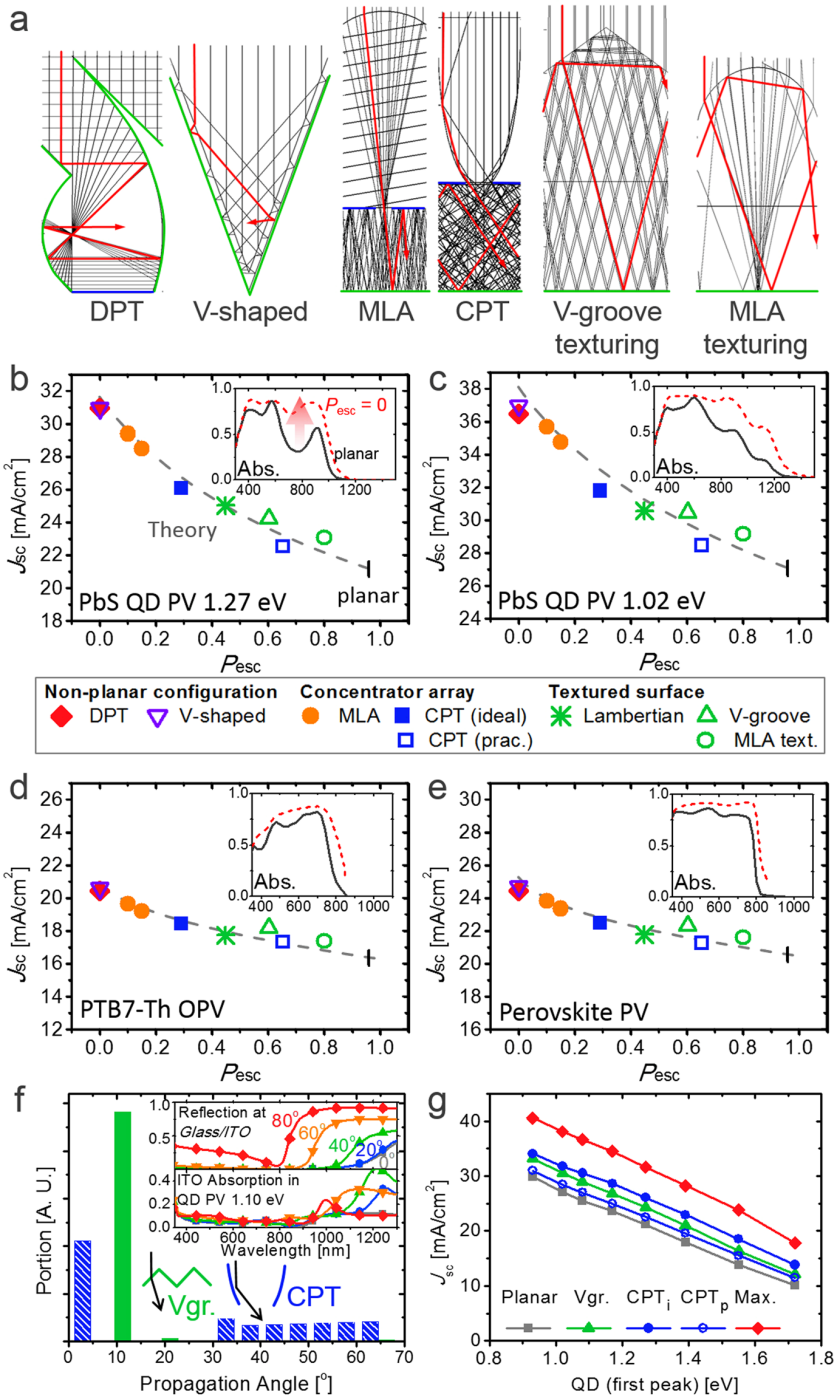
(**a**) Ray-traced images of the unit structure of periodic arrays for the various light trapping schemes. Green and blue lines indicate PVs and metal mirrors, respectively^38,45,49^. (**b**–**e**) Simulated values for the schemes of (**a**) and calculated *J*_sc_ values (dashed line) according to *P*_esc_ of (**b**) QD PV with PbS 1.27 eV, (**c**) QD PV with PbS 1.02 eV, (**d**) PTB7-Th OPV, and (**e**) perovskite PV. (**f**) Distribution of the photon propagation angle inside the substrate after passing V-groove texturing (green) and CPT (blue). (inset: Fresnel reflection ratio at the interface between glass and ITO (above) and parasitic absorption of ITO in a full device using QD 1.10 eV with various propagation angles). (**g**) Calculated *J*_sc_ values of QD PVs with various bandgaps and schemes of V-groove texturing, ideal CPT (CPT_i_), practically modified CPT (CPT_p_), and maximum with *P*_esc_ = 0.

Figure 3a exhibits the ray-traced images for the representative ray-optical schemes that suppress *P*_esc_. DPT, consisting of two parabolas sharing one focal point, has been proposed as an ideal configuration to realize *P*_esc_ = 0^49^. Folded configurations such as V-shape can also induce high *N*_bounce_ by directing incident photons to the next PV^49,51,52,54^. Concentrator arrays such as a micro lens array (MLA)^47,49^ or compound parabolic trapper (CPT)^4^ block the internal photons by implementing blocking mirrors between the entrances. The textured surfaces with a V-groove^39,44,45^ or MLA shape^38,43^ can be considered as a practical scheme to reduce *P*_esc_ by total internal reflection (TIR).

The performance of those schemes for diverse PVs is summarized in Fig. 3b–e. For the gray theoretical lines, short-circuit current densities (*J*_sc_) were obtained by integrating the active absorption spectra for the normal propagation angle according to *P*_esc_, where the internal quantum efficiency (IQE) was calculated by comparing the integrated active absorption and experimentally obtained *J*_sc_ values (IQE = 1 for QD and perovskite PVs and 0.95 for OPV), ignoring its spectral dependency and MEG for simplicity. The estimated *J*_sc_ values of the light trapping schemes are plotted as a function of *P*_esc_ by employing a custom-made multiscale optical simulation^4,45,49^. Whereas the DPT and V-shaped (vertex angle of 40°) structures mostly show the lowest *P*_esc_ (~0) and the highest *J*_sc_ enhancements, their utilization may be limited by their relatively complicated manufacturing processes (i.e., nonplanar structures)^49^. MLA^47^ and CPT^4^ with blocking mirrors also show low *P*_esc_ and excellent performance. Whereas MLA suffers from limited angular performance^49^, CPT uses compound parabolic concentrators (CPCs) to secure the sufficient acceptance angle with a reasonable *P*_esc_ of 0.292. On the other hand, the practically modified CPT with no blocking mirrors and a truncated CPC height, as described in the previous report^4^, shows relatively low performance (*P*_esc_ = 0.653). Compared to these schemes, attaching textured films to the top surface is simpler. A Lambertian scattering surface can realize, as theoretically dictated, *P*_esc_ equal to 1/*n* ^2^ ^13,45,61,62^. The V-groove^39,45^ and MLA^38^ textured surfaces have relatively higher *P*_esc_; however, their performance was above the theoretical line because their geometries can also work as an antireflection (AR) structure, reducing the Fresnel reflection loss (~4%) on the substrate^4,45^.

Although the simulated results for those light trapping schemes are approximately matched with the theoretical values for given *P*_esc_ values, the general tendency was shown to be different for each PV. The maximum *J*_sc_ enhancement for *P*_esc_ = 0 is 26.5% for PTB7-Th(poly[[4,8-bis[(2-ethylhexyl)oxy]benzo[1,2-b:4,5-b′]dithiophene-2,6-diyl][3-fluoro-2-[(2-ethylhexyl)carbonyl]thieno[3,4-b]thiophenediyl]])-based OPV and 22.8% for perovskite PV, and it becomes relatively larger for QD PVs (40.6% for 1.02 eV and 49.3% for 1.27 eV) as greater room for enhancement exists as shown in the insets of Fig. 3b–e.

Notably, for QD PVs with low bandgaps, the performance of DPT, V-shaped, CPT, and Lambertian structures is shown to be lower than the theoretical expectation from their *P*_esc_ values. Those schemes have a relatively high level of light scattering compared to schemes such as MLA and V-groove texturing as shown in Fig. 3f. Although ITO is a transparent material for visible light, it becomes metallic for NIR photons as shown in the refractive indices of Figure S1. Therefore, surface reflection and parasitic absorption loss of ITO^4,13,45^ become significant at long wavelengths with large propagation angles as shown in the inset of Fig. 3f. Therefore, the schemes with large angle scattering result in reduced performance within the NIR region. Figure 3g shows that the difference between V-groove texturing and practical CPT increases for lower-bandgap QD PVs having more NIR absorption. Those issues have not been previously considered because large-bandgap PVs such as OPVs and perovskite PVs are not influenced by the optical characteristics in the NIR region. In addition to the tradeoff between voltage and current characteristics along the bandgap, such optical characteristics and potential for a light trapping effect should also be considered when designing QD PV systems.

By considering both practicality and effectiveness, we selected CPT (practical) and V-groove textures for experimental comparison. Moreover, VCPT^4,50^ combining CPT with a V-groove textured surface is also considered. The structures were realized by attaching microstructured PDMS (polydimethylsiloxane, *n* = 1.42) films with the same structures as in the previous reports^4,45,50^. For QD PVs, we chose PbS QD with a bandgap of 1.27 eV as an active material to achieve the best efficiency.

For a conventional PV measurement system, a current density (*J*)–voltage (*V*) curve is obtained under AM 1.5 G illumination with an aperture of a defined area to prevent the overestimation of photocurrent emanating out of the cell area. However, although this system is considered as a standard for planar PVs, it is possible to underestimate the photocurrent where a scattering layer is embedded on the topside because scattered light may deviate from the cell area. For that reason, many previous studies^4,38,40,43,45^ for ray-optical light trapping structures have chosen measurement without apertures, but such measurement issues have been rarely examined.

**Figure 4:**
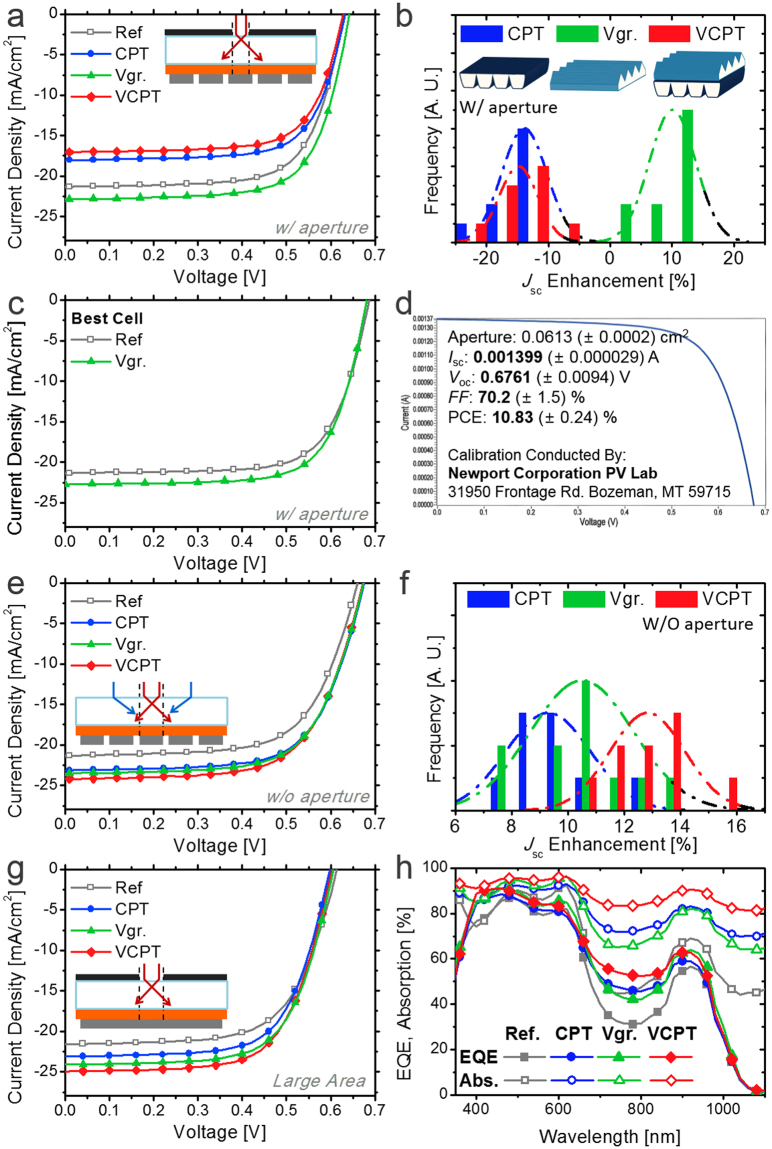
(**a**,**e**) *J*–*V* characteristics and (**b**,**f**) *J*_sc_ enhancements of QD PV with light trapping schemes (**a**,**b**) with and (**e**,**f**) without aperture. (**c**) *J*–*V* characteristics of the best cell with aperture with and without V-groove texturing. (**d**) Certified data for V-groove textured device of (**c**) (Newport Co.). (**g**) *J*–*V* characteristics of large-area QD PV with light trapping schemes and aperture. (h) EQE and absorption spectra of PV in (**g**). (EQE-integrated *J*_sc_ = 22.6 (ref.), 24.8 (CPT), 25.4 (Vgr.), and 26.3 (VCPT) mA/cm^2^, respectively).

Figure 4a and Table 1 show the *J*–*V* characteristics of the QD PV with and without light trapping schemes in the standard measurement system. An aperture with a defined illumination area of 0.0625 cm^2^ was applied for the device with 5 cells of 0.12–0.17 cm^2^ size in parallel as shown in Figure S2a. The PCE of the control device was 9.28% with a spectral mismatch factor of 0.930. Whereas the best PCE of 10.2% (*J*_sc_ 7.1% ↑) was achieved with the V-groove textured film, the *J*_sc_ values and PCEs for CPT and VCPT devices were even much lower than the reference. Such current reduction mainly results from the optical loss in the measurement setup using apertures. Whereas light through the aperture is fully illuminated inside the cell area for flat control devices, that with scattering surfaces may deviate from the cell and be lost as depicted in the inset of Fig. 4a. Compared to CPT and VCPT with high-level scattering angles, such optical loss was relatively small for V-groove texturing, so the average *J*_sc_ enhancements of 10.1% could be achieved from repeated measurements as shown in Fig. 4b.

**Table 1:** Photovoltaic characteristics using ray-optical schemes.

System	Scheme	*J*_*sc*_ [mA/cm^2^]	Δ*J*_*sc*_ [%]	*V*_*oc*_ [V]	*FF* [%]	*η* [%]
w/aperture (0.0613 cm^2^)	Ref.	21.3		0.629	69.1	9.28
	CPT	18.0	−15.6	0.632	70.4	8.01
	V-gr.	22.9	7.1	0.641	69.5	10.2
	VCPT	17.1	−20.1	0.628	69.6	7.45
	Best Ref.	21.4		0.687	70.0	10.3
	Best V-gr.	22.8	6.7	0.682	70.5	11.0
w/o aperture (0.167 cm^2^)	Ref.	21.3		0.661	65.3	9.21
	CPT	23.2	8.6	0.675	66.3	10.4
	V-gr.	23.5	10.2	0.674	65.9	10.5
	VCPT	24.2	13.6	0.673	64.9	10.6
Large area (0.350 cm^2^)	Ref.	21.6		0.613	64.9	8.60
	CPT	23.1	7.1	0.598	66.6	9.20
	V-gr.	24.1	11.7	0.605	66.5	9.71
	VCPT	25.0	15.6	0.602	65.6	9.86

In this study, we used only the middle cell of the 5 fingers on each device to secure the periodic boundary condition, which will be described in Figs 4e and S2a. On the other hand, along with the cell-by-cell deviation of the efficiency, the highest efficiency greater than 10% was observed in an edge-side cell of one device. By attaching V-groove textured film, the efficiency of the best cell was improved from 10.3% to 11.0% (*J*_sc_ 6.7% ↑) as shown in Fig. 4c. The result shows that the light trapping effect can be consistently achieved even for highly efficient devices. Our best device was certified by Newport Corporation, and the PCE was 10.83(±0.24)% with V-groove texturing as shown in Figs 4d, and S5. During the certification, the V-groove textured film may have been contaminated by repeated processes of attaching and detaching. We noticed that the PCE of the retrieved device was recovered by replacing the film with a fresh one that had 3% increased *J*_sc_.

Underestimation of the light trapping effect can be minimized by full illumination on finger-type PVs without apertures as depicted in the inset of Figs 4e and S2a–d. In that case, only the middle cell among 5 fingers can be measured to assume the periodic boundary condition (PBC), so the amount of light scattered from the outside to the cell (blue) is considered to be the same as that scattered from the cell to the outside (red), ignoring the optical loss through the gaps between the cells. We characterized the PV performance in that system. We used an effective cell area, which is calculated by dividing the total photocurrent by *J*_sc_ from the same device with an aperture, rather than the geometrical cell area, to exclude the possible overestimation factors such as underestimation of the cell area or charge diffusion from outside^4^. In that system with the same device used in Fig. 4a, PCE was improved from 9.21% to 10.4% (*J*_sc_ 8.5% ↑), 10.5% (*J*_sc_ 10.2% ↑), and 10.6% (*J*_sc_ 13.6% ↑) by attaching CPT, V-groove texturing, and VCPT films, respectively, as shown in Fig. 4e and Table 1. Figure 4f clearly shows that the highest *J*_sc_ enhancement is achieved by VCPT (12.8% on average) when the aperture is removed, whereas that for V-groove texturing (10.5% on average) is not much different from that with the aperture.

Obviously, the most accurate evaluation of the light trapping effect can be achieved in devices with large areas and a sufficient marginal distance (>5 mm) as depicted in the inset of Figs 4g and S2e–h. Apparently, the large-area configuration is closer to the practical outdoor modules in terms of optics. As shown in Fig. 4g and Table 1, the PCE of a large-area (2.10 cm^2^) QD PV with an aperture of 0.350 cm^2^ was improved from 8.60% to 9.20% (*J*_sc_ 7.1% ↑), 9.71% (*J*_sc_ 11.7% ↑), and 9.86% (*J*_sc_ 15.6% ↑) by adopting CPT, V-groove texturing, and VCPT, respectively. Conversely, the *J*_sc_ enhancements of finger-type cells without an aperture (Fig. 4e,f) are 1–2% lower. Whereas the small optical loss may result from the 1 mm gaps between the 3 mm fingers (Figure S2a), the measured enhancements are much closer to those of the practical large-area devices than those of small-area devices with apertures (Fig. 4a,b), supporting the validity of our method.

The fabrication of large-area devices enables achievement of the spectral responses without optical loss^4^. The measured external quantum efficiencies (EQEs) and total device absorption (=1 − *reflection*) are shown in Fig. 4h. Each value was measured in both polarizations to remove the polarizing effect of the light source. The EQE was enhanced by adopting light trapping schemes over the whole spectral range, but the enhancements were not uniform owing to the spectral characteristics as discussed in Fig. 3f. EQE data for the lower-bandgap (1.17 eV) QD PV are shown in Figure S3 to evaluate the spectral influence of light trapping schemes.

## Conclusion

Optical schemes including plasmonic nanostructures, nonplanar configurations (DPT and V-shaped), concentrator arrays (MLA and CPT), and textured surfaces (Lambertian, V-groove, and MLA) have been studied to improve QD PV efficiency. The certified PCE of the best cell was improved from 10.3% to 11.0% by light trapping, and enhancement greater than 15% was proved to be possible for practical large-area devices with VCPT.

## Materials and Methods

The performance of the ray-optical schemes was evaluated by a custom-made multiscale simulation, which was used for our previous studies^4,40,45,49^. Two hundred rays with a full spectrum were traced for each unit structure, and the PBC was applied.

For device fabrication, ZnO synthesized by the sol-gel method was spin-coated onto ITO substrates at 3000 rpm for 30 s and annealed at 200 °C for 10 min. PbS QD films (270 nm) were deposited using a layer-by-layer (LBL) spin-coating process on the ZnO substrate at 2500 rpm as described in previous reports^30,31^. For the LBL spin-coating process, solid-state ligand exchange was performed using 7 mg mL^−1^ of 1-ethyl-3-methylimidazolium iodide (EMII) solutions (in methanol) and 0.01% ethanedithiol (EDT) solutions (in acetonitrile) on the surface of the PbS films. After the LBL spin-coating process, the sample was stored in nitrogen environment for 12 h, and post-annealing was performed at 100 °C for 10 min. Finally, Au (70 nm) layers were deposited by thermal evaporation. *J*–*V* characteristics of PVs were obtained under 1 sun illumination with the AM 1.5 G spectrum using a K201 LAB55 (McScience, Korea) solar simulator. *J*–*V* curves were swept in the reverse direction, and the hysteresis was shown to be negligible (<1%). A spectral mismatch factor of 0.93 was applied. Finger-type devices with a cell area of 0.12–0.17 cm^2^ and aperture of 0.0613 cm^2^ were used for the experiments. All light-trapped devices were measured immediately after the reference devices to fairly compare the *J*_sc_ enhancement. To secure a sufficient number of data points for *J*_sc_ enhancements in Fig. 4b,f, old devices, which have the same structure and possibly include degradation, were also used for the experiment.

The datasets generated during and/or analysed during the current study are available from the corresponding author on reasonable request.

## Electronic supplementary material

Supporting information
