# Supplementary material for: Broadband light trapping strategies for quantum-dot photovoltaic cells (>10%) and their issues with the measurement of photovoltaic characteristics
Source: Sci Rep. 2017 Dec 12;7:17393. doi: 10.1038/s41598-017-17550-4 (PMC5727208; doi:10.1038/s41598-017-17550-4)
Supplement: Supplementary file 1 — Supporting information [file 41598_2017_17550_MOESM1_ESM.doc]

Supplementary information

Broadband light trapping strategies for quantum-dot photovoltaic cells (>10%) and their issues with the measurement of photovoltaic characteristics

Changsoon Cho^1^, Jung Hoon Song^2^, Changjo Kim^1^, Sohee Jeong^2^, *, and Jung-Yong Lee^1^, *

*^1^*Graduate School of Energy, Environment, Water, and Sustainability (EEWS), Korea Advanced Institute of Science and Technology (KAIST), Daejeon 34141, Republic of Korea, *^2^*Institute of Machinery & Materials (KIMM), Daejeon 34103, Republic of Korea, *e-mail: sjeong@kimm.re.kr, jungyong.lee@kaist.ac.kr

Optical constants used for the simulations


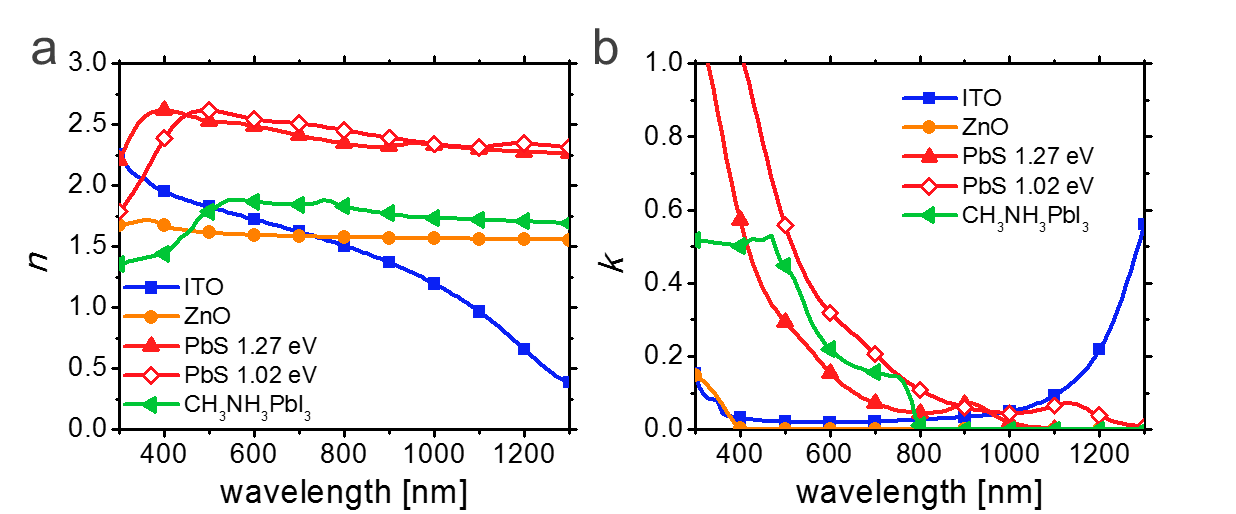


Figure S1 (a) Real and (b) imaginary parts of refractive indices used in the simulation

The refractive indices of ITO, ZnO, PbS, and CH_3_NH_3_PbI_3_ are shown in Figure S1. Those of ZnO and PbS 1.27 eV were obtained using ellipsometry; that of PbS 1.02 eV was modified from PbS 1.27 eV; and those of ITO^1^ and CH_3_NH_3_PbI_3_^2^ were obtained from the literature. The refractive indices were assumed to be isotropic. Bandgaps of PbS were determined by Tauc plot with *n* = 2.^3,4^

**Details for absorption enhancement with respect to *P*_esc_**

The performance of the generalized ray-optical light trapping schemes on thin-film PVs can be analyzed using *P*_esc_ of the internal photons (Figure 2c). If internal reflections occur on the optical components, *P*_esc_ decreases, and the rays can have more chances to be absorbed by the PV layers at the bottom. For a fixed propagation angle and specific wavelength, the maximum absorption by active materials (i.e., *P*_esc_ = 0) is given as

, (1)

where *A*_act_ is the absorption efficiency of the active layer per bounce of internal incident light; *A*_parasitic_ is the parasitic absorption of layers other than the active layer in the PV; and *T*_inc_ is the transmittance of the incident light through the optical component. This formula indicates the maximum absorption that can be obtained at a designated propagation angle and with perfect light trapping schemes. To realize maximal absorption, *P*_esc_ should be as close to zero as possible; *N*_bounce_ becomes infinite in this case. The fraction of absorption of active layers to parasitic absorption per bounce then determines the maximum absorption efficiency as derived above. The *intrinsic* loss, *A*_parasitic_, is generally small in reference unit cells; however, the total parasitic absorption loss becomes nonnegligible as the total absorption increases by efficient light trapping. We can achieve the maximal active absorption, which is close to *T*_inc_, by suppressing *A*_parasitic_ as much as possible in perfect light trapping schemes.

However, in general, for a practical case with a nonzero acceptance angle range, *P*_esc_ cannot be zero. Therefore, the absorption expressed in (1) cannot really be achieved, and it should be revised as

, (2)

where *A*_ref_ is an optical loss that occurs when the ray is internally reflected once on the optical component. *A*_ref_ is zero for total internal reflection on the top surface; however, it becomes nonzero when reflecting metal is established, as in the blocking mirrors of CPT. It should be noted that for a regular planar cell, both *T*_inc_ and *P*_esc_ are ~0.96 at normal incidence because of Fresnel reflection on the glass substrate surface. With the above equation, we can easily estimate the active absorption of a light-trapped cell according to the cell structure and *P*_esc_ without considering the absorption variation with the propagation angle change. For Figure 2d and 3b–e, *A*_ref_ was assumed to be zero, and *T*_inc_ was assumed to be 0.96.

Cell configurations for light trapping assessment


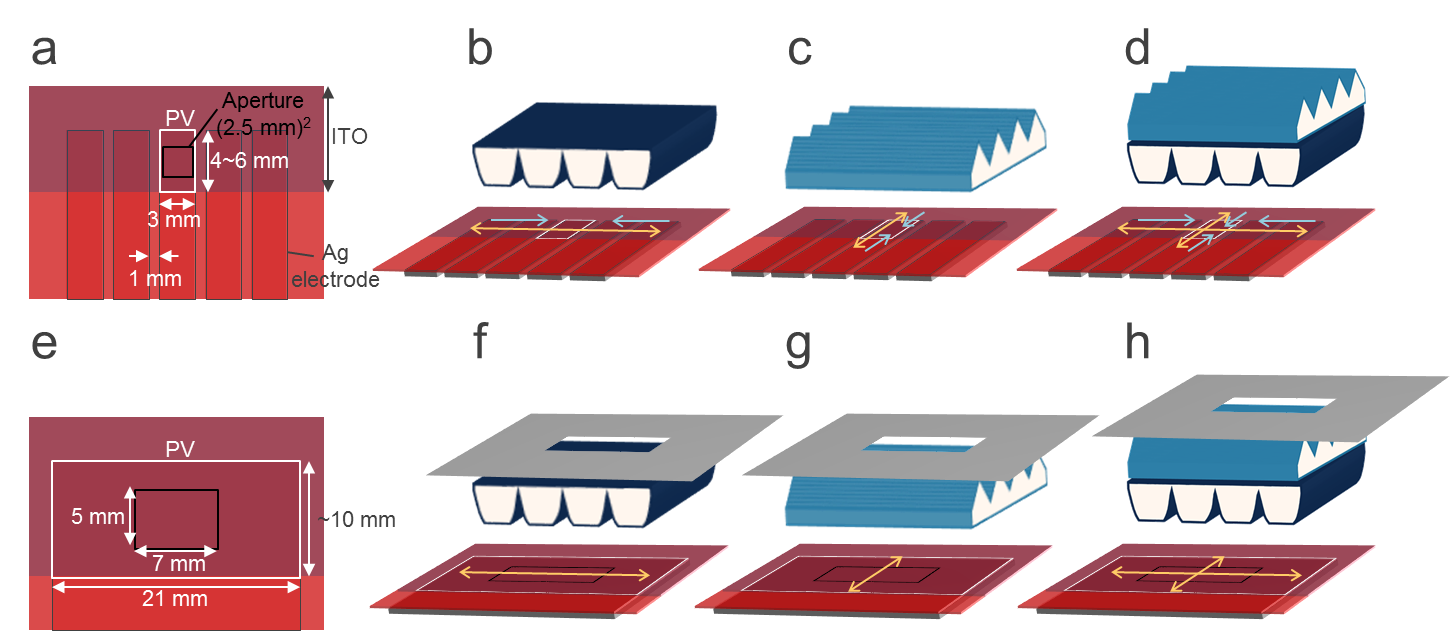


Figure S2 Configurations of (a–d) finger-type and (e–h) large-area devices with dimensions shown in (a, e). (b, f), (c, g), and (d, h) represent the PV measurements with CPT, V-groove texturing, and VCPT, respectively.

Figure 4e–f (finger-type) and Figure 4g (large area) were obtained as shown in Figure S2a–d and Figure S4e-h, respectively, whereas Figure 4a–d were obtained by adopting the aperture on the configurations of Figure S2a–d. The aperture of 2.5 mm × 2.5 mm size has a marginal distance of 2–3 mm on the PV area when light is scattered in the vertical direction. Although the average *J*_sc_ enhancement of 10.1% is achieved by V-groove texturing with such a small margin, a significant portion of incident light is shown to deviate from the PV area in the system using CPT or VCPT with large scattering. Therefore, as shown in Figure S2b, CPT is positioned to scatter light in the horizontal direction, and *J*_sc_ was measured without the aperture by assuming the periodic boundary condition that the amount of light deviating from the cell (orange) is the same as the amount of light coming from outside to the PV area (blue). Practically, in CPT and VCPT, the optical loss of light coming from outside through the gaps (= 1 mm) between the patterns of the Ag electrode possibly results in the underestimation of the CPT performance. On the other hand, in large-area devices shown in Figure S2e–h, the aperture has a margin of 14 mm and 5 mm in the horizontal and vertical directions, respectively, so *J*_sc_ enhancements of ~7.1% and ~15.6% are achieved by CPT and VCPT, respectively.

Bandgap and thickness dependency of QD PVs


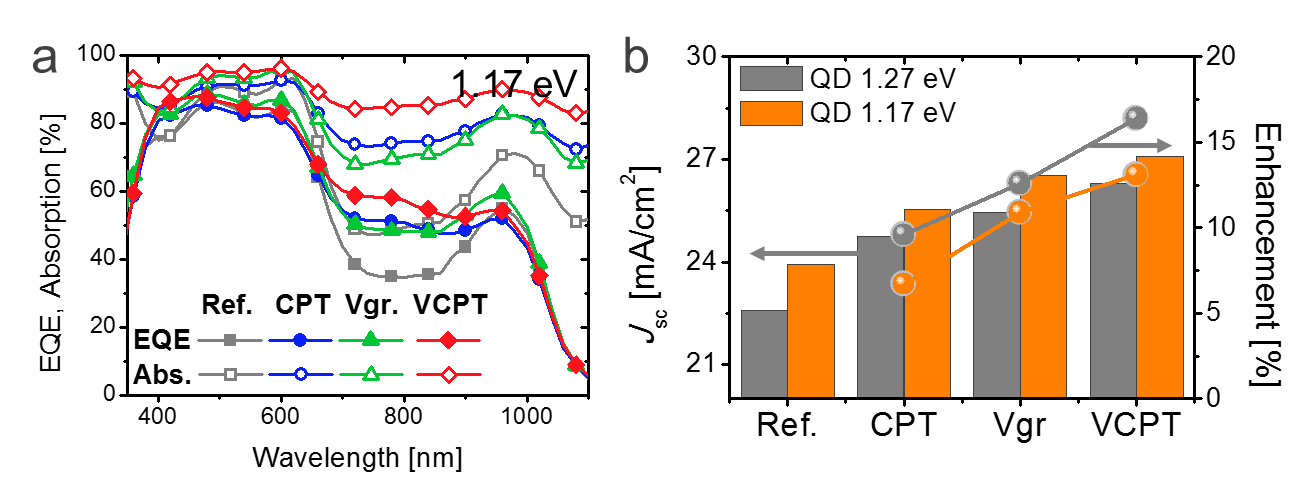


**Figure S3** (a) EQE and absorption spectra of large-area QD PV (1.17 eV) with light trapping schemes. (b) Photocurrent densities integrating EQE and enhancements of light trapping schemes for QD PVs with 1.27 eV and 1.17 eV.

To experimentally examine the relationship between the light trapping effect and QD bandgap, the large-area QD PV with a reduced bandgap (1.17 eV) was fabricated with other fabrication conditions maintained. Compared to the QD PV with a bandgap of 1.27 eV shown in Figure 4h, the EQE and absorption spectra are extended to a longer wavelength as shown in Figure S3a. In this low-bandgap device, light trapping characteristics in the NIR region are more clearly exhibited. Whereas only the V-groove texturing enhances the EQE for a whole spectrum, EQEs of CPT and VCPT are even lower than that of the control device for the wavelength longer than 930 nm and 960 nm, respectively. Those characteristics support our assertion that a large propagation angle is not beneficial for QD PVs in the NIR region due to the increased reflection and parasitic absorption of ITO as shown in Figure 3f. Because the relative amount of NIR photon absorption increases as the QD bandgap decreases, the overall enhancement of *J*_sc_, obtained by integrating EQE, is shown to be relatively low for CPT and VCPT in QD PV of 1.17 eV, compared to those for 1.27 eV, as shown in Figure S3b.

Supporting data for the measurement


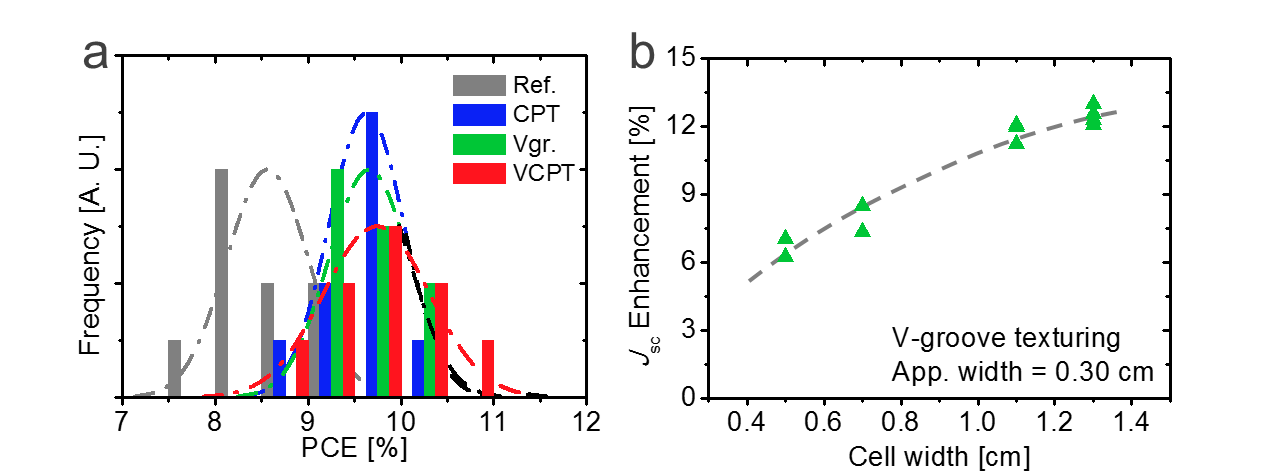


Figure S4 (a) Histogram for PCE of QD devices with and without light trapping used in Figure 4e and 4f. (b) Measured *J*_sc_ enhancement of V-groove texturing as a function of cell width for a given aperture with a width of 0.30 cm.

For the experiments, we measured the performance of >10 old and fresh devices without an aperture. On average, by applying CPT, V-groove texturing, and VCPT, PCE was improved from 8.56(±0.44)% to 9.63(±0.41)%, 9.66(±0.44)%, and 9.74(±0.57)%, respectively; *J*_sc_ was improved from 20.2(±0.6) mA/cm^2^ to 22.1(±0.6) mA/cm^2^, 22.4(±0.7) mA/cm^2^, and 22.8(±0.8) mA/cm^2^, respectively; *V*_oc_ was slightly increased from 0.659(±0.011) V to 0.669(±0.011) V, 0.668(±0.012) V, and 0.665(±0.012) V, respectively, due to the increased current density; and *FF* was only changed from 64.2(±1.4)% to 65.1(±1.1)%, 64.4(±1.2)%, and 64.1(±1.2)%, within the error ranges, respectively. The light trapping effects have been consistently achieved regardless of the reference efficiencies as shown in Figure 4f. We also examined the influence of the cell geometry on *J*_sc_ enhancement. With a fixed aperture width of 0.30 cm, the cells with various areas were measured with V-groove texturing. *J*_sc_ enhancement of V-groove texturing is then shown to increase as the cell width and corresponding marginal distance increase as shown in Figure S4b. Where the cell width becomes > 1.1 cm (marginal distance > 0.8 cm), the measured enhancement becomes similar to that for the large-area device in Figure 4g. The measurements for Figure 4a and 4b were performed for the small-area devices with a marginal distance of 2–3 mm, so the *J*_sc_ enhancements were not as high as those for a large area, and the deviation was shown according to their area, which is determined by ITO half-etching area.

Certification of efficiency


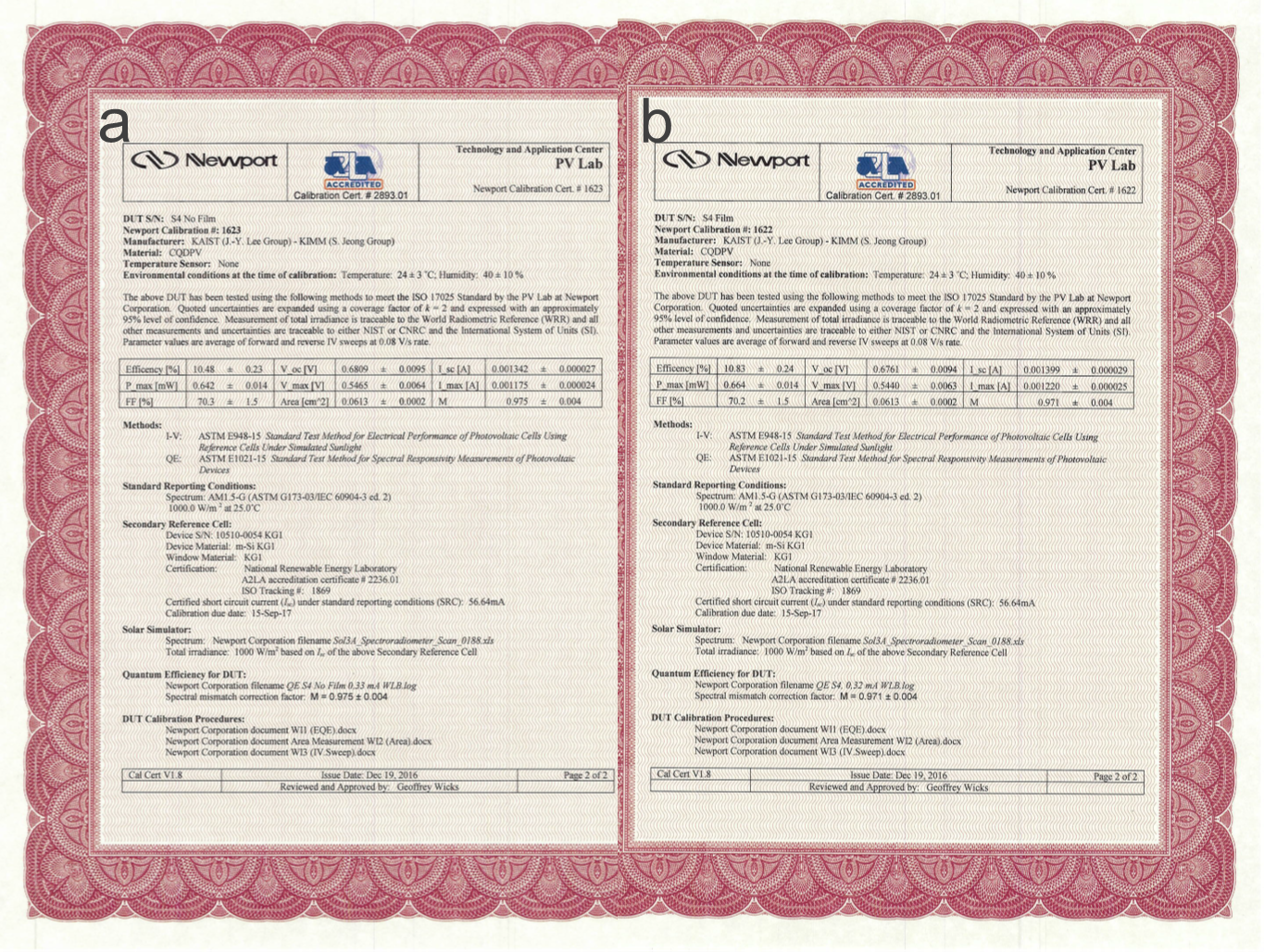


Figure S5 Photovoltaic cell calibration certificate of QD PVs (a) without and (b) with V-groove texturing. (device size = 0.123 cm^2^; aperture area = 0.0613 cm^2^)

The best efficiencies were certified by Newport Corporation as shown in Figure S5. PCE was improved from 10.5% to 10.8% and *J*_sc_ was 4.2% improved by attaching V-groove texturing on the best device for the certification. During the certification, a single V-groove film was re-used multiple times and possibly contaminated. After the certification, we measured the efficiency of the returned device to which the same used film was attached, and we confirmed that *J*_sc_ was 3% increased additionally by replacing the film with a fresh one.

Calculation using transfer-matrix formalism (TMF)


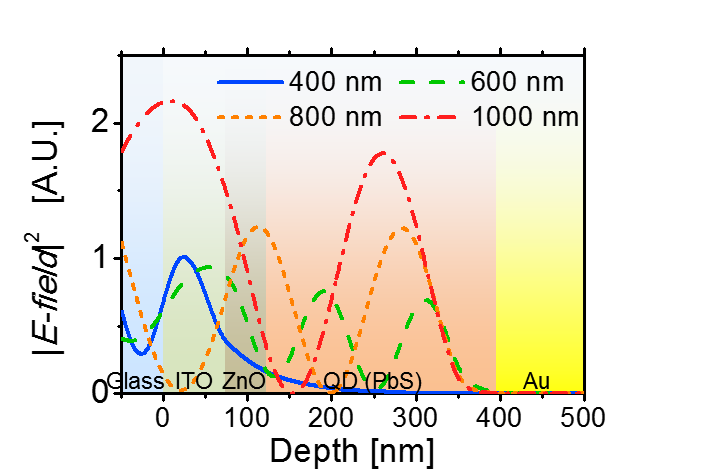


Figure S6 Calculated internal profile of |*E-field*|^2^ inside a QD PV (1.27 eV) at various wavelengths

TMF is a simple and well-known method to calculate the optical properties of planar PVs.^5,6^ We calculated the profiles of internal electric and magnetic fields inside the multilayers using transfer-matrices at each layer and interface as shown in Figure S6. Then, the spatial rate of energy dissipation is proportional to |*E*(*x*)|^2^ and the absorption of active layer can be easily obtained by integrating the energy dissipation for each wavelength as described in the previous literatures^5,6^. While the absorption simply increases as the layer thickness increases in a bulk-film model, light absorption can be sensitive to the variation of internal field profiles in those sub-wavelength scale structures. Figure 2a shows that the absorption peaks are changed by increasing the thicknesses and it can be attributed to the modified interference effects inside the sub-wavelength-thick QD layer.

Quality of light trapping structures


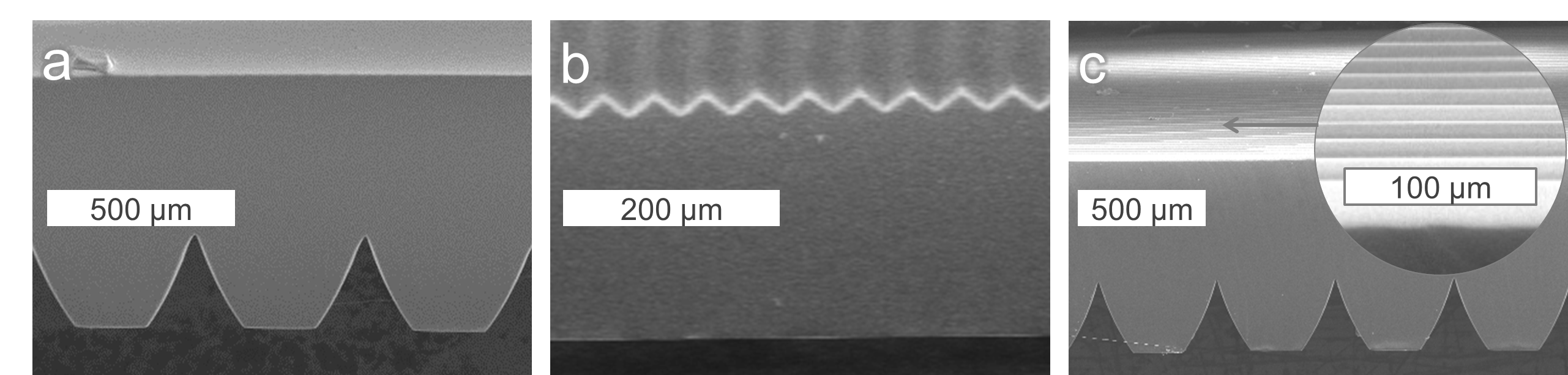


Figure S7 Scanning electron microscope (SEM) images of (a) CPT, (b) V-groove texturing, and (c) VCPT structures.

Figure S7 shows cross-sectional images of light trapping structures. Those films are fabricated with the same molds used for our previous reports^1, 7^. The films followed the designed geometries well and their surface was smooth. According to our previous studies^1, 7^, thickness of the film does not make a difference in their light trapping effects. However, as we discussed in Figure 4a, light scattering can cause a problem of underestimation for the standard measurement system, and therefore, the V-groove texturing film was prepared to be thin (~200 μm) to minimize the lateral propagation of light. For CPT, the film thickness was not highly controlled since the incident light propagates just normally before meeting parabolic structures. When they were applied to PVs, we were careful not to have any bubbles and dusts between the film and substrate. The films are easily contaminated and the performance is decreased when the film is re-used on PV; hence, we used fresh films for every measurement in our laboratory.

Absorbance of quantum-dots

Figure S8 Measured absorbance spectrum of PbS quantum-dots we used.

Figure S8 shows the absorbance spectrum of PbS quantum-dots in solution, which we used for the experiments. The first excitonic peak appears near ~850 nm (1.45 eV) and its calculated bandgap is 1.27 eV.

References

1 Cho, C. *et al.* Toward Perfect Light Trapping in Thin-Film Photovoltaic Cells: Full Utilization of the Dual Characteristics of Light. *Adv. Optical Mater.* 3, 1697-1702 (2015).

2 Chang, S.H., Lin, K.F., Chiang, C.H., Chen, S.H. & Wu, C.G.. Plasmonic structure enhanced exciton generation at the interface between the perovskite absorber and copper nanoparticles. Sci. World J., 128414-128419 (2014).

3 Gao L.F. *et al.*, Small molecule-assisted fabrication of black phosphorus quantum dots with a broadband nonlinear optical response. Nanoscale 8, 15132-15136 (2016).

4 Woomer A.H. *et al.*, Phosphorene: Synthesis, Scale-Up, and Quantitative Optical Spectroscopy. Acs Nano 9, 8869-8884 (2015).

5 Pettersson, L. A. A., Roman, L. S. & Inganas, O. Modeling photocurrent action spectra of photovoltaic devices based on organic thin films. *J. Appl. Phys.* 86, 487-496 (1999).

6 Peumans, P., Yakimov, A. & Forrest, S. R. Small molecular weight organic thin-film photodetectors and solar cells. *J. Appl. Phys.* 93, 3693-3723 (2003).

7 Cho, C. *et al.* Random and V-groove texturing for efficient light trapping in organic photovoltaic cells. *Sol. Energy Mater. Sol. Cells* 115, 36-41 (2013).
